# Prone position during venovenous extracorporeal membrane oxygenation: survival analysis needed for a time-dependent intervention

**DOI:** 10.1186/s13054-022-03923-3

**Published:** 2022-02-08

**Authors:** Akram M. Zaaqoq, Adrian G. Barnett, Silver Heinsar, Matthew J. Griffee, Graeme MacLaren, Jeffrey P. Jacobs, Jacky Y. Suen, Gianluigi Li Bassi, John F. Fraser, Heidi J. Dalton, Giles J. Peek, the COVID-19 Critical Care Consortium (COVID Critical)

**Affiliations:** 1 Department of Critical Care Medicine, MedStar Washington Hospital Center, Georgetown University, 110 Irving St NW, office 4B-65, Washington, DC 20010 USA; 2 School of Public Health and Social Work, Queensland University of Technology, Brisbane, QLD Australia; 3 Critical Care Research Group, Faculty of Medicine, University of Queensland and The Prince Charles Hospital, Brisbane, Australia; 4 Department of Anesthesiology, University of Utah School of Medicine, Salt Lake City, UT USA; 5 Cardiothoracic Intensive Care Unit, National University Hospital, National University of Singapore, Singapore, Singapore; 6 Congenital Heart Center, Shands Children’s Hospital, University of Florida, Gainesville, FL USA; 7 Institut d’Investigacions Biomediques August Pi i Sunyer, Barcelona, Spain; 8 Adult Intensive Care Services, The Prince Charles Hospital, Brisbane, Australia; 9 Department of Pediatrics, Inova Fairfax Hospital, Falls Church, VA USA

## To the Editor,

We read with great interest the paper of Giani and colleagues titled "Prone positioning during venovenous extracorporeal membrane oxygenation for acute respiratory distress syndrome: a pooled individual patient data analysis" published in the critical care journal [1]. We are surprised that their meta-analysis failed to show a survival benefit for prone positioning during venovenous extracorporeal membrane oxygenation (VV ECMO). We would like to postulate that this was due to the limitation of the statistical methods.

Prone positioning patients with moderate to severe acute respiratory distress syndrome (ARDS) for an extended time during their illness has been shown to reduce their 28 and 90-day mortality [2]. This is thought to be due to a combination of improved ventilation/perfusion matching, better distribution of transpulmonary pressures, reduced pulmonary vascular resistance, and right ventricular afterload [3]. In addition, respiratory system compliance is improved directly through enhanced lung compliance and indirectly through reducing chest and abdominal wall pressure [4]. More importantly, prone positioning may reduce ventilator-induced lung injury (VILI) [3]. Despite these benefits, prone ventilation remains underutilized; only 33% of patients in the APRONET trial, a large multicenter study of patients, with severe ARDS were placed in prone position [5].

The same physiologic benefits for prone positioning have been shown during VV ECMO support for severe ARDS. Despite this, observational outcome studies are conflicting, showing both improved and worsened survival. In an observational analysis of 25 ECMO patients with COVID-19 severe ARDS, prone positioning showed improved oxygenation but a higher mortality rate which was attributed to the severity of illness [6]. Meta-analysis is a standard technique to reduce differences between treatment groups by increasing sample size. However, Giani and colleagues did not find an improvement in outcome with prone positioning during VV ECMO. We believe this could be due to failing to consider the temporal properties of prone ventilation, despite an adequate sample size of 889 patients. Our survival analysis of 232 VV ECMO patients in the Coronavirus Disease 2019 Critical Care Consortium international registry showed prone positioning during ECMO was associated with a reduced probability of death (hazard ratio, 0.31; 95% CI 0.14–0.68) [7]. Our results are consistent with the meta-analysis of 1836 patients from thirteen studies, which showed prone positioning of VV ECMO patients with severe ARDS led to reduced mortality at 28, 60-, and 90-days [8].

This inconsistency could be a failure to address the fact that prone positioning has temporal dimensions, having both a duration and a time course in the patient's illness journey. We used a multistate survival model to address this issue, which is a more realistic model of patient progression through their journey [9]. Patients in prone positioning can move to the supine state and vice versa, and this transition contributes to the complexity of the model and eventually affects the outcome [10]. Hazard ratios from the survival model provide an estimate for transitions between states. A survival approach can account for the average time spent in the prone position, the number still at risk over time, and the probability of transitions [11]. It can also account for the effect of multiple confounders on the transition and outcomes while accounting for the key confounder of time prone positioning began. Analyzing time-dependent treatments using cross-sectional groups that ignore time (e.g., "any prone during stay" and "no prone during stay") can cause significant biases in the effects of the treatment [12]. These cross-sectional comparisons are confounded by time in ICU, which predicts both the probability of receiving prone treatments, and key outcomes such as death and length of stay.

In conclusion, we believe that prone positioning is beneficial for VV ECMO patients with Covid-19 ARDS and continue to recommend its use. Consideration of the temporal aspects of prone ventilation such as day of initiation, time of day of proning, the duration of prone positioning, and number of proning episodes is essential in both retrospective analyses and future randomized controlled clinical trials.

## Data Availability

Not applicable.

## References

[1] Giani M, Rezoagli E, Guervilly C, Rilinger J, Duburcq T, Petit M, Textoris L, Garcia B, Wengenmayer T, Grasselli G (2022). Prone positioning during venovenous extracorporeal membrane oxygenation for acute respiratory distress syndrome: a pooled individual patient data analysis. Crit Care (Lond, Engl).

[2] Guérin C, Reignier J, Richard J-C, Beuret P, Gacouin A, Boulain T, Mercier E, Badet M, Mercat A, Baudin O (2013). Prone positioning in severe acute respiratory distress syndrome. N Engl J Med.

[3] Richard JC, Bregeon F, Costes N, Bars DL, Tourvieille C, Lavenne F, Janier M, Bourdin G, Gimenez G, Guerin C (2008). Effects of prone position and positive end-expiratory pressure on lung perfusion and ventilation. Crit Care Med.

[4] Pelosi P, Tubiolo D, Mascheroni D, Vicardi P, Crotti S, Valenza F, Gattinoni L (1998). Effects of the prone position on respiratory mechanics and gas exchange during acute lung injury. Am J Respir Crit Care Med.

[5] Guérin C, Beuret P, Constantin JM, Bellani G, Garcia-Olivares P, Roca O, Meertens JH, Maia PA, Becher T, Peterson J (2018). A prospective international observational prevalence study on prone positioning of ARDS patients: the APRONET (ARDS Prone Position Network) study. Intensive Care Med.

[6] Garcia B, Cousin N, Bourel C, Jourdain M, Poissy J, Duburcq T (2020). Prone positioning under VV-ECMO in SARS-CoV-2-induced acute respiratory distress syndrome. Crit Care (Lond, Engl).

[7] Zaaqoq AM, Barnett AG, Griffee MJ, MacLaren G, Jacobs JP, Heinsar S, Suen JY, Bassi GL, Fraser JF, Dalton HJ et al. Beneficial effect of prone positioning during venovenous extracorporeal membrane oxygenation for coronavirus disease 2019. Crit Care Med. 2021. 10.1097/CCM.0000000000005296 PMC8796833 34582415

[8] Papazian L, Schmidt M, Hajage D, Combes A, Petit M, Lebreton G, Rilinger J, Giani M, Le Breton C, Duburcq T et al. Effect of prone positioning on survival in adult patients receiving venovenous extracorporeal membrane oxygenation for acute respiratory distress syndrome: a systematic review and meta-analysis. Intensive Care Med. 2022. 10.1007/s00134-021-06604-x PMC8762989 35037993

[9] Nasri MR, Reza M (2007). Global stability of a deterministic model for HIV infection in vivo. Chaos Solitons Fract.

[10] Matsena Zingoni Z, Chirwa TF, Todd J, Musenge E (2021). A review of multistate modelling approaches in monitoring disease progression: Bayesian estimation using the Kolmogorov-Chapman forward equations. Stat Methods Med Res.

[11] Jackson C (2011). Multi-state models for panel data: the msm Package for R. J Stat Softw.

[12] Beyersmann J, Gastmeier P, Wolkewitz M, Schumacher M (2008). An easy mathematical proof showed that time-dependent bias inevitably leads to biased effect estimation. J Clin Epidemiol.

